# Adverse physiological effects of smoking cessation on the gastrointestinal tract: A review

**DOI:** 10.1097/MD.0000000000035124

**Published:** 2023-09-22

**Authors:** Mueataz A. Mahyoub, Sarah Al-Qurmoti, Ayesha Akram Rai, Mustafa Abbas, Majed Jebril, Mohammed Alnaggar, Shuixiang He

**Affiliations:** a Department of Gastroenterology, Faculty of Medicine and Health Sciences, Thamar University, Dhamar, Yemen; b Department of Gastroenterology, First Affiliated Hospital of Xi’an Jiaotong University, Xi’an, China; c Clinical Medical Research Center for Digestive Diseases (Oncology) of Shaanxi Province, Xi’an, China; d Department of Cleft Palate-Craniofacial Surgery, College of Stomatology, Xi’an Jiaotong University, Xi’an, Shaanxi, China; e School of Medicine, Xi’an Jiaotong University, Xi’an, China; f Department of Internal Medicine, Faculty of Medicine and Health Sciences, Thamar University, Dhamar, Yemen; g College of Health Sciences, Department of Laboratory Medical Sciences, The Islamic University of Gaza, Gaza, Palestine; h Department of Internal Medicine, Clinic Medical College, Hubei University of Science and Technology, Xianning, Hubei, China; i Department of Oncology, South Hubei Cancer Hospital, Xianning, Hubei, China.

**Keywords:** adverse physiological effects of smoking cessation, clinical professionals, gastrointestinal tract

## Abstract

Smoking cessation is known to have numerous health benefits, but it can also induce adverse physiological effects, including those affecting the gastrointestinal tract (GIT). Understanding the adverse physiological effects of smoking cessation on the GIT is critical for healthcare professionals and smokers attempting to quit, as it enables them to anticipate and manage potential challenges during the smoking cessation process. Although the detrimental effects of smoking on the GIT have been well established, there is a gap in the literature regarding the specific physiological reactions that may occur upon smoking cessation. This mini-review summarizes the current literature on the predisposing factors, pathophysiology, clinical presentation, and treatment options for adverse physiological effects of smoking cessation on the GIT. We aimed to raise awareness among busy clinical professionals about these adverse effects, empowering them to effectively support individuals striving to quit smoking and maintain their cessation. By consolidating the existing knowledge in this field, this review offers practical implications for smokers, healthcare providers, and policymakers to optimize smoking cessation interventions and support strategies to improve health outcomes.

## 1. Introduction

Smoking is a dangerous habit that has severe consequences for an individual’s health and digestive system, including Crohn disease, functional dyspepsia, gastroesophageal reflux disease, chronic pancreatitis, and various types of gastrointestinal cancers such as those affecting the esophagus, stomach, colon, pancreas, and liver.^[1,2]^ Tobacco smoke consists of more than 7000 chemicals, among which at least 70 are known to be carcinogenic. The harmful chemicals in tobacco smoke, such as tar, carbon monoxide, formaldehyde, benzene, acetone, cadmium, and lead, can interfere with the normal functioning of the digestive system.^[3,4]^ Consequently, smoking could slow digestion, reduce the production of digestive juices, and cause chronic acid reflux.^[5]^

When a person ceases smoking, the digestive system slowly functions normally again. However, during the initial cessation phase, the individual may experience temporary adverse physiological effects of smoking cessation on the gastrointestinal tract (APESCGIT).^[6]^ The APESCGIT presentations may be uncomfortable, but they usually indicate that the body adapts to the absence of nicotine and other harmful chemicals from tobacco smoke. As the body fully adjusts, the APESCGIT presentations should subside, and the digestive system should return to normal.^[7]^

Despite the initial APESCGIT, quitting smoking offers numerous long-term benefits.^[8]^ Improved overall health, reduced risk of chronic diseases such as cardiovascular and respiratory diseases, and enhanced quality of life due to better respiratory function, increased energy levels, and improved physical fitness are significant advantages of quitting smoking.^[9]^ Therefore, encouraging smokers to quit is crucial for their overall well-being, and clinical professionals must offer continual support throughout the quitting process.^[10]^ This review summarizes the current literature on the predisposing factors, pathophysiology, clinical presentations, and treatment of APESCGIT. We aimed to raise awareness among busy clinical professionals about APESCGIT, empowering them to effectively support individuals striving to quit smoking and maintain their cessation. By consolidating the existing knowledge, this review offers practical implications for smokers, clinical professionals, and policymakers to optimize smoking cessation interventions and support strategies to improve health outcomes.

## 2. Predisposing factors

The predisposing factors for developing APESCGIT in smoking cessation individuals (nicotine dependence consequences) are multifactorial and encompass various sociodemographic, behavioral, environmental, individual, and biological factors.

Sociodemographic factors, such as younger age and male gender, have been found to be positively correlated with a higher risk of APESCGIT in smoking cessation individuals compared to older age and female gender.^[11–15]^ Moreover, the history and frequency of smoking, along with other substances like alcohol, marijuana, and illicit drugs, have been identified as critical predisposing factors for developing APESCGIT in smoking cessation individuals.^[16,17]^ Individual traits such as psychiatric disorders, delinquency, and a tendency to seek novelty are also significant predictors of APESCGIT after smoking cessation.^[18]^ Environmental factors, including exposure to smokers in the immediate social environment, such as parents and peers, may further increase the susceptibility for APESCGIT in smoking cessation individuals.^[19]^ Finally, biological factors such as initial sensitivity to nicotine, exposure to smoking during prenatal development, nicotine metabolism, and genetic susceptibility have been linked to the development of APESCGIT in smoking cessation individuals.^[14,20]^

## 3. Pathophysiology

The pathophysiology of APESCGIT is a multifaceted process that involves various mechanisms. These mechanisms include changes in the digestive system, alterations in the gut microbiota, changes in the nervous system, and psychological factors. Firstly, smoking cessation can lead to changes in the digestive system due to nicotine withdrawal. These changes include decreased gastric acid secretion, increased gastric emptying, and alterations in gut motility.^[21]^ Secondly, the gut microbiota is crucial in maintaining digestive health, and smoking cessation could alter its composition.^[22]^ Improving taste and smell senses after quitting smoking could result in changes in dietary habits, impacting the gut microbiota.^[23]^ Thirdly, smoking cessation could affect the nervous system, specifically the enteric nervous system, which regulates digestive function. Nicotine withdrawal could lead to dysregulation of the enteric nervous system. Lastly, psychological factors like stress could impact the digestive system during smoking cessation. Stress could cause changes in gut motility, alter the gut microbiota and play a role in developing oral ulcers.^[24–28]^

The presynaptic nicotinic acetylcholine receptors (nAChRs) on the terminal region of myenteric neurons play an important role in the modulation of gastrointestinal motility.^[29]^ Withdrawal symptoms from nicotine use (APESCGIT) are largely attributed to the activity of nAChRs, the primary targets for nicotine and the endogenous neurotransmitter acetylcholine. Chronic nicotine exposure could lead to neuroadaptations that affect various neurotransmitter systems, including dopamine, glutamate, gamma-aminobutyric acid, and serotonin.^[30]^ It has been suggested that the desensitization and upregulation of nAChRs resulting from chronic nicotine use contribute to the manifestation of APESCGIT after quitting smoking.^[31–33]^ Additionally, sustained nAChR desensitization may play a crucial role in alleviating nicotine withdrawal in humans.^[34]^

Studies have shown that cortisol can stimulate the secretion of ghrelin, a hormone that promotes appetite and food consumption. Therefore, decreased cortisol levels following smoking cessation may reduce ghrelin secretion, leading to decreased appetite.^[35–37]^ On the other hand, nicotine has appetite-suppressing effects that can lead to weight loss by reducing food intake, increasing energy expenditure, boosting the resting metabolic rate, and promoting lipolysis and fat oxidation. However, the withdrawal of nicotine’s impact on the central nervous system remains the primary reason for weight gain following smoking cessation.^[38,39]^

## 4. Clinical presentation

**Figure 1.**
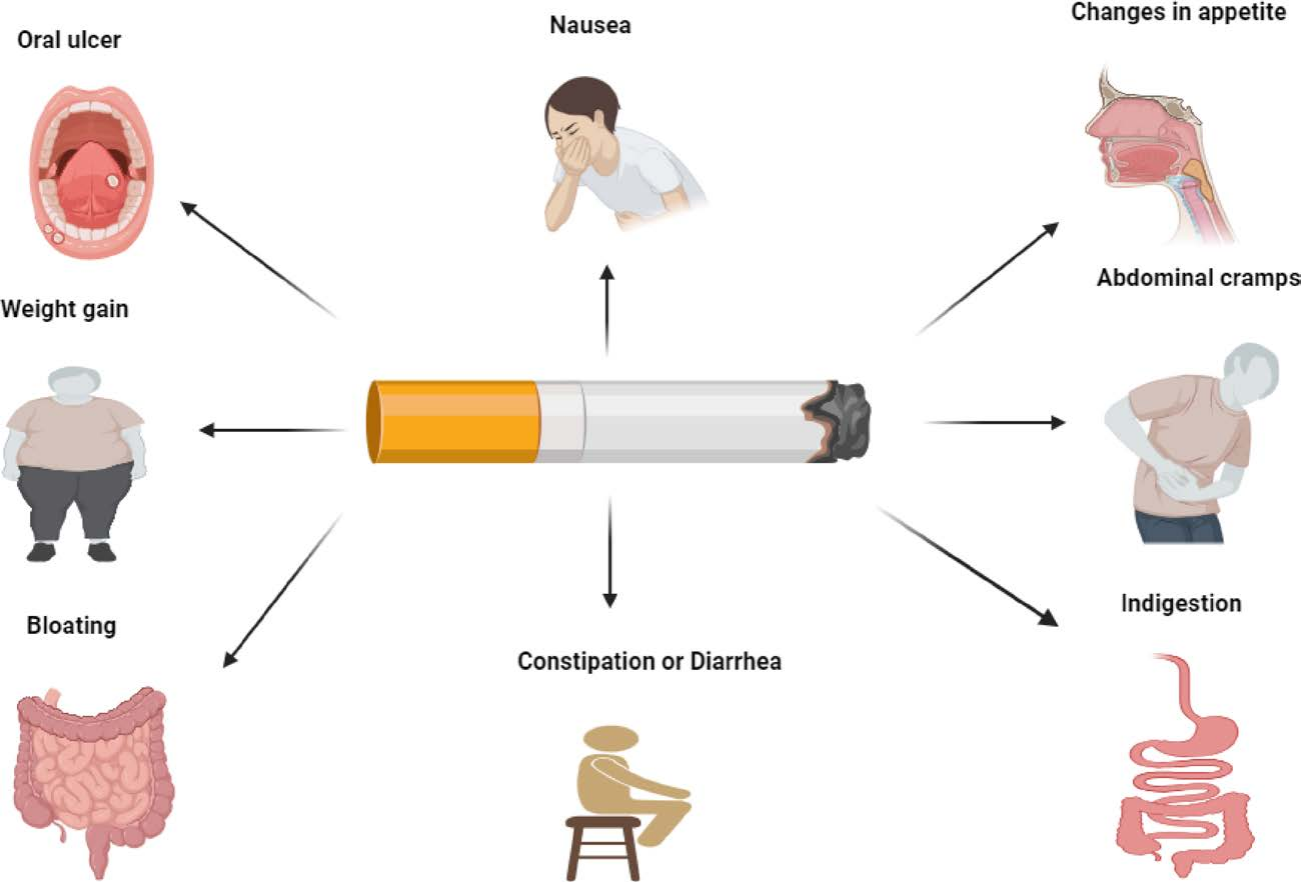
The presentations of APESCGIT. The change of appetite could be hunger or anorexia. APESCGIT = adverse physiological effects of smoking cessation on the gastrointestinal tract.

The APESCGIT presentations could range from mild to severe. Some of the APESCGIT presentations are opposite to the effects of nicotine use.^[40]^ However, some APESCGIT presentations are not the opposite of nicotine effects. They may present as oral ulcers, changes in appetite, nausea, abdominal cramps, indigestion, bloating, weight gain, constipation, and or diarrhea (Fig. 1).^[41–45]^

Some patients may experience oral ulcers during smoking cessation, as reported in a study involving 1234 smokers.^[44,46]^ The study found that 40% of patients experienced oral ulcers after quitting smoking, with a higher prevalence observed in more dependent smokers. Most of these ulcers developed within the first 2 weeks of quitting and resolved within 4 weeks in 60% of patients. Although generally mild, 8% of patients reported severe ulcers, and ratings were higher in patients using oral nicotine replacement products than in other cessation methods during the first week of abstinence. Patients must be reassured that these oral ulcers during smoking cessation are temporary and not a side effect of cessation medications. The occurrence of oral ulcers while quitting smoking may be attributed to anxiety and emotional stress from nicotine withdrawal.^[47]^

The relationship between smoking cessation and appetite is multifaceted and subject to individual variation. Numerous studies have observed increased appetite upon quitting smoking, which has been attributed to alterations in leptin and ghrelin levels.^[48,49]^ However, other studies have reported a decrease in appetite, particularly noticeable in the second week following smoking cessation.^[50]^ The underlying reasons for this decrease in appetite remain unclear, although they may be associated with changes in taste and smell perception, heightened metabolism, and psychological influences.^[51]^

M.M. Ward et al^[52]^ the incidence of transient nausea among individuals who have ceased smoking is relatively low in absolute terms. However, it is 4 times higher in proportion than those who have never smoked. Additionally, the mean severity rate of the smoking cessation effect for nausea remained consistent from the first day until day 28. Nausea is an APESCGIT presentation that individuals frequently encounter during their smoking cessation process when quitting smoking. It can result from various factors, including the withdrawal effects of nicotine and other chemicals found in cigarettes and physiological adjustments that the body undergoes as it adapts to smoke-free.^[53]^

Abdominal cramps, indigestion, and bloating are frequent in smoking cessation individuals. A cohort study revealed that former smoking was found to be associated with functional bloating, with an odds ratio of 1.18 and a 95% confidence interval ranging from 1.04 to 1.33.^[54]^

Ceasing smoking is linked with an overall increase in weight. The variations between individuals who quit and those who keep smoking vary from 2.6 to 5.3 kg, indicating a notable difference in the weight gained.^[55]^ A meta-analysis of 62 clinical trials on smoking cessation discovered that abstaining for a year is linked to an average weight gain of 4 to 5 kg. Most of this weight gain typically occurs during the first 3 months of quitting, with a slowing rate of increase after that.^[56]^

An increase in constipation often accompanies the cessation of smoking. A study has demonstrated that constipation exhibits a moderate yet statistically significant association with other markers of tobacco withdrawal, affecting approximately 17% of individuals who undergo smoking cessation, with a notable impact on 9% of quitters. Despite a potential reduction in severity after 2 weeks of abstinence, constipation may persist even after 4 weeks of quitting.^[57]^

It is important to note that APESCGIT presentations are temporary and tend to improve over time as the body adjusts to the absence of nicotine and other substances in tobacco smoke.^[58]^ However, some individuals may experience more severe symptoms that could significantly affect their quality of life. The duration of APESCGIT could vary from person to person, depending on various factors, such as smoking history, overall health, stress levels, age, and gender.^[24]^

Smoking history is a crucial factor in the severity of APESCGIT. Heavy smokers who have smoked for an extended period are more likely to experience severe APESCGIT than those who smoked fewer cigarettes or for a shorter duration.^[59,60]^ Additionally, individuals with preexisting digestive disorders or a weakened immune system may experience more severe APESCGIT. Stress and anxiety could also exacerbate APESCGIT in smoking cessation individuals, with individuals who experience high-stress levels being more susceptible to severe AESCGIT.^[61]^ Furthermore, age and gender could also influence APESCGIT severity in smoking cessation individuals, with some studies suggesting that women may experience more severe APESCGIT than men. Typically, APESCGIT in smoking cessation individuals are most severe in the first week after quitting and begin to improve in the subsequent weeks. However, some individuals may continue to experience APESCGIT for several months or even longer, especially those with a long smoking history.^[25,26,52,60,62–69]^

Notably, several factors could exacerbate APESCGIT during smoking cessation. A diet high in processed foods, sugar, and fat can contribute to APESCGIT and worsen them.^[70]^ A sedentary lifestyle can also slow digestion and worsen APESCGIT in smoking cessation individuals.^[71]^ Alcohol could irritate the digestive system and exacerbate APESCGIT; caffeine can stimulate the digestive system and worsen APESCGIT.^[25,72–74]^

## 5. Treatment

Smoking cessation could cause 1 or more APESCGIT presentations that negatively affect the quality of life. Therefore, effective treatment of APESCGIT is crucial to support a successful transition to a smoke-free life. Treating APESCGIT could involve a combination of lifestyle changes and medications. Incorporating lifestyle modifications could be an effective strategy for managing the APESCGIT. A balanced diet with high-fiber foods such as fruits, vegetables, and whole grains could help relieve constipation and improve overall digestive health.^[75]^ Regular exercise could stimulate the digestive system and alleviate bloating and abdominal pain.^[76]^ Many studies found that exercise can have similar effects to smoking in terms of stimulating the central nervous system and neurobiological processes in the brain.^[77]^ This includes increasing beta-endorphin levels, which are also increased in smokers. As a result, some experts suggest that exercise could be a substitute for smoking as a reinforcer.^[78–82]^

Stress-management techniques such as deep breathing, meditation, or yoga could also help improve overall digestive health by reducing stress.^[83]^ Sometimes, lifestyle modifications may not be enough to manage APESCGIT, and medications may be necessary. Antacids like calcium carbonate or magnesium hydroxide could help neutralize stomach acid and relieve heartburn. Laxatives, including fiber supplements and stool softeners, could help relieve constipation. Proton pump inhibitors could help reduce stomach acid and relieve symptoms of acid reflux.^[47,84–88]^

## 6. Conclusion

APESCGIT could range from mild to severe and may present as oral ulcers, changes in appetite, nausea, abdominal cramps, indigestion, bloating, weight gain, constipation, and or diarrhea. The severity of the APESCGIT is a better predictor of unsuccessful smoking attempts than smoke intake or dependence. Treating APESCGIT could be a combination of lifestyle modifications and medications. Incorporating a balanced diet, regular exercise, and stress-management techniques could help alleviate symptoms such as constipation, bloating, and abdominal pain. Medications such as antacids, laxatives, and proton pump inhibitors could also relieve some cases. The increase in awareness among clinical professionals could effectively support individuals trying to quit smoking and aid them in maintaining their cessation.

## Author contributions

**Conceptualization:** Mueataz A. Mahyoub.

**Project administration:** Mueataz A. Mahyoub.

**Supervision:** Mueataz A. Mahyoub, Shuixiang He.

**Writing – original draft:** Mueataz A. Mahyoub.

**Writing – review & editing:** Sarah Al-Qurmoti, Ayesha Akram Rai, Mustafa Abbas, Majed Jebril, Mohammed Alnaggar, Shuixiang He.
